# Handbook of Diseases of the Skin

**Published:** 1885-07

**Authors:** 


**Handbook of Diseases of the Skin.** Edited by H. Von Ziemssen, M.D., Professor of Clinical Medicine in Munich, Editor of "Ziemssen's Cylopædia." Illustrated with 80 wood engravings, and color prints. New York: Wm. Wood & Co. 1885.

The subscribers to "Ziemssen's" have here a gift of no small value. The work will take rank with the best treatises on dermatology. The article from the pen of the distinguished Professor Unna on the "Anatomy and Development of the Skin and its Appendages," is one of the best on the subject which has appeared in any language. The other contributors to the volume are all men distinguished in this special field. Space will not allow us to call attention to the merits of the different contributions, but the work as a whole will be highly appreciated by its fortunate possessors. We are not informed whether the volume is for sale to those who may desire it alone, but it ought to be, for the work is too valuable to be confined only to those who are subscribers to " Ziemssen's." In another column we call attention to the request of the publishers, that all those to whom the present volume was promised, will send them their present address together with their address at the time they subscribed.

